# Dietary L-Ornithine L-Aspartate Alleviates Ammonia Load and Improves Hepatic Health in Largemouth Bass (*Micropterus salmoides*)

**DOI:** 10.3390/ani16142190

**Published:** 2026-07-14

**Authors:** Lizhen Jia, Guangzhi Ma, Shiwei Xie, Beiping Tan, Shuyan Chi, Junming Deng

**Affiliations:** 1 College of Fisheries, Guangdong Ocean University, Zhanjiang 524088, China; jlz18276758998@163.com (L.J.); xswzsdx@163.com (S.X.); bptan@126.com (B.T.); chishuyan77@163.com (S.C.); 2 Anhui Wanhe Jial Biotechnology Co., Ltd., Chuzhou 239000, China; linxiangqin2025@163.com

**Keywords:** L-ornithine L-aspartate, protein metabolism, antioxidant, immunity, largemouth bass

## Abstract

The intensive culture of largemouth bass (*Micropterus salmoides*) under high-protein feeding conditions is frequently associated with ammonia nitrogen stress, which adversely affects hepatic health. L-Ornithine L-aspartate (LOLA) has been proposed as a regulator of nitrogen metabolism. However, its effects in aquafeeds remain unclear. Five experimental diets (LOLA0, LOLA3, LOLA6, LOLA9, LOLA12) containing graded levels of LOLA (0%, 0.03%, 0.06%, 0.09%, 0.12%) were formulated, along with a diet supplemented with 0.09% L-ornithine + L-aspartate (OA9). Each diet was fed to fish reared in triplicate cages (30 fish per cage, initial body weight: 7.38 ± 0.02 g) for 10 weeks. No significant effects were observed on growth performance (weight gain, specific growth rate) or feed utilization (feed conversion rate, protein efficiency ratio) among the different LOLA treatments (*p* > 0.05). Nevertheless, second-order polynomial regression analysis revealed that dietary inclusion of 0.05–0.08% LOLA significantly reduced plasma ammonia and urea nitrogen levels, decreased hepatic aspartate aminotransferase and glutamate dehydrogenase activities, as well as plasma adenosine monophosphate deaminase activity, activated the hepatic TOR-S6K1-related markers, and reduced mortality under ammonia nitrogen stress (*p* < 0.05). Furthermore, supplementation with 0.07–0.08% LOLA significantly enhanced glutathione peroxidase and superoxide dismutase activities in both serum and liver, as well as serum levels of lysozyme and immunoglobulin M. Additionally, compared with the OA9 group, the LOLA9 group showed more pronounced changes in TOR-S6K1-related markers, ammonia load, antioxidant and immune parameters. Overall, while no significant growth enhancement was observed, these biochemical and physiological modifications suggest that LOLA supplementation is associated with alterations in nitrogen metabolism, antioxidant, and immune indices in largemouth bass, but further studies are needed to confirm the functional significance of these changes.

## Simple Summary

High-protein feeding in intensive largemouth bass aquaculture often causes ammonia nitrogen stress, impairing hepatic health. L-Ornithine L-aspartate (LOLA) regulates nitrogen metabolism, but its effects in aquafeeds are unclear. We formulated five experimental diets containing graded levels of LOLA (0%, 0.03%, 0.06%, 0.09%, 0.12%), along with a diet supplemented with 0.09% L-ornithine + L-aspartate (OA9). After a 10-week feeding trial, the results showed that moderate LOLA supplementation at approximately 0.05–0.08%, as determined by second-order polynomial regression analysis, effectively reduced ammonia and urea nitrogen levels in the blood, enhanced liver antioxidant capacity and immune function, and improved survival under acute ammonia stress. Moreover, LOLA was more effective than the equivalent mixture of free ornithine and aspartate. These findings suggest that dietary LOLA may serve as a promising feed additive to improve liver health and alleviate ammonia-related stress in intensive aquaculture systems.

## 1. Introduction

Largemouth bass (*Micropterus salmoides*) has become a globally important aquaculture species because of its desirable flesh quality and rapid growth performance [1]. According to statistics reported for 2024, production in China reached 939,000 tons, representing a 5.68% year-over-year increase [2]. However, the intensive culture of this species often relies on high-protein feed to achieve rapid growth [3]. For juvenile largemouth bass, dietary protein levels of approximately 47% crude protein have been shown to significantly increase total ammonia-nitrogen (TAN) concentrations in culture water compared with lower protein diets (42% or 44%), indicating that this level imposes a measurable ammonia excretion burden [4]. While meeting nutritional requirements, high-protein feeding can induce ammonia nitrogen stress through multiple interacting mechanisms. Specifically, dietary protein in excess of growth requirements is catabolized as an energy source, leading to increased endogenous ammonia production via amino acid deamination [5]. Along with this endogenous production, intensive systems with high stocking densities result in the accumulation of uneaten feed and metabolic waste, contributing to elevated environmental ammonia concentrations. This environmental ammonia, in turn, can impair branchial ammonia excretion capacity, further increasing internal ammonia accumulation and exacerbating toxicity [6,7,8]. It should be noted, however, that whether high-protein feeding results in pathological hyperammonemia depends on multiple factors, including the magnitude of protein excess, the duration of exposure, and the fish’s capacity to maintain nitrogen homeostasis. Under balanced dietary conditions, fish can effectively dispose of excess nitrogen through normal excretory pathways without developing ammonia toxicity. The liver is the primary site for protein metabolism, ammonia detoxification, and energy metabolism in fish [9]. In this context, it is important to note that fish species differ substantially in their nitrogen excretion strategies and ureagenic capacity. For largemouth bass, urea-N accounts for only about 30% of total nitrogen excretion under normal conditions [10]. Moreover, under ammonia stress, the largemouth bass liver predominantly converts ammonia into glutamine via glutamine synthetase, with urea synthesis playing a relatively minor role [11]. A sustained hyperammonemic state further impairs the body’s capacity to clear ammonia by exacerbating hepatic injury, thereby creating a vicious cycle. Therefore, identifying feed additives that enhance hepatic health is crucial for addressing the challenges associated with high-protein, high-density farming of largemouth bass.

L-Ornithine L-Aspartate (LOLA), an endogenous amino acid compound composed of ornithine and aspartate, has been shown in numerous clinical trials to regulate nitrogen metabolism and the urea cycle in patients with liver cirrhosis or hepatic encephalopathy (HE) following intravenous or oral administration [12,13,14]. Additionally, research on the application of LOLA in the treatment of liver diseases such as non-alcoholic fatty liver disease (NAFLD) has progressed substantially, although most studies have been conducted in animal models such as rats and mice [15,16,17]. In recent years, the potential application of LOLA in aquatic animal health has garnered increasing attention. Preliminary studies indicate that LOLA may exert beneficial effects by alleviating hyperammonemia and ammonia toxicity in fish, thereby improving hepatic health. By modulating inflammatory mediators and influencing serum transaminase activity, LOLA reduces blood ammonia levels, thereby enhancing systemic ammonia clearance and metabolism [18]. Studies suggest that LOLA may have greater efficacy than separate supplementation of ornithine and aspartate, although its mechanism of action remains unclear. Therefore, the primary objective of this study was to investigate whether dietary LOLA supplementation could alleviate ammonia-related metabolic stress and improve hepatic health in largemouth bass. Secondary objectives included evaluating its effects on growth performance, protein metabolism, antioxidant capacity, and immune responses, as well as comparing the efficacy of LOLA with an equivalent mixture of free ornithine and aspartate. The findings are expected to provide a theoretical basis and technical support for the application of LOLA in aquaculture.

## 2. Materials and Methods

### 2.1. Experimental Diets

Six isonitrogenous and isolipidic experimental diets were formulated, supplemented with 0%, 0.03%, 0.06%, 0.09%, or 0.12% LOLA, or with 0.09% of a mixture of L-aspartic acid and L-ornithine hydrochloride (Table 1). Since no information is available on the appropriate supplementation level of LOLA in aquafeeds, the dosage range was designed with reference to a previous study on low-dose dietary N-carbamoylglutamate (NCG) supplementation in juvenile yellow catfish (0.025%) [19]. Considering that both LOLA and NCG are key functional amino acids involved in the urea cycle, this range was adopted as a reference. The LOLA supplementation levels were set in a geometric series (0.03%, 0.06%, 0.09%, 0.12%) to allow for dose–response evaluation. To enable an equimolar comparison with the LOLA9 group (0.09% LOLA), the OA9 diet was formulated to contain 0.09% of a mixture of L-aspartic acid and L-ornithine hydrochloride, with inclusion levels calculated on an equimolar mass basis (1 mol LOLA = 1 mol L-ornithine + 1 mol L-aspartate; 0.09% LOLA corresponds to 0.045% L-aspartate and 0.057% L-ornithine hydrochloride). All additives were supplied by Anhui Wanhe Jial Biotechnology Co., Ltd. (Chuzhou, China). The experimental diets were prepared according to the method described by Chen et al. [20]. The pellets were then dried in a temperature-controlled chamber at 25 °C and stored at −20 °C until use.

### 2.2. Fish Management

An outdoor 10-week feeding trial from 17 June to 27 August 2024 was conducted in cement ponds (5 m × 5 m × 1.2 m) with net cages supplied with aerated groundwater at Guangdong Ocean University. Water temperature, pH, and dissolved oxygen were maintained at 24–30 °C, 7.0–8.0, and >6.0 mg/L, respectively. The temperature variation reflects natural diurnal and weather-related fluctuations inherent to outdoor rearing conditions; all experimental cages were located within the same pond and thus exposed to identical environmental conditions. Juvenile fish were obtained from Helian Aquaculture Farm (Maoming, China). Each treatment included three replicate cages (1.2 m × 1.0 m × 0.8 m). Healthy fish (initial body weight: 7.38 ± 0.02 g) were randomly distributed into 18 cages, and the cages were randomly allocated to each dietary treatment. Each cage was considered an independent experimental unit (*n* = 3 per treatment). Fish were fed twice daily at 08:00 and 17:00.

### 2.3. Sample Collection

At the end of the 10-week feeding trial, after a 24-h fasting period, the total biomass and number of fish in each cage were recorded to calculate growth performance. Fish were anesthetized with eugenol diluted at 1:10,000. Blood was collected from the caudal vein of six fish per cage, centrifuged at 3500 rpm for 10 min, and the resulting serum from these six fish was pooled within the same cage before storage at −80 °C until analysis. In addition, hepatic tissues from three separate fish per cage were excised and pooled per cage for the analysis of protein metabolism, antioxidant capacity, and immune responses. Furthermore, an additional three liver samples per cage were immersed in RNAlater solution and pooled per cage before storage at −80 °C for mRNA expression analysis. For each pooled cage sample, three technical replicate measurements were performed and averaged. All data were analyzed using cage means (*n* = 3 per treatment).

### 2.4. Analysis

#### 2.4.1. Proximate Composition

Nutrient profiling of the diets was conducted according to the AOAC method [21]. Moisture content was calculated from weight loss after drying at 105 °C until a constant weight. Crude protein was quantified using a KjeltecTM8400 analyzer (FOSS, Höganäs, Sweden), crude lipid content was determined by Soxhlet extraction with petroleum ether, and ash content was measured by incineration at 550 °C for 6 h.

#### 2.4.2. Protein Metabolism-Related Parameters

Plasma levels of total amino acids (TAA), blood urea nitrogen (BUN), blood ammonia (BA), insulin (INS), growth hormone (GH), and insulin-like growth factor 1 (IGF-1), as well as the activities of adenosine monophosphate deaminase (AMPD) and glutamate dehydrogenase (GDH), were measured using ELISA kits supplied by Shanghai Enzyme-linked Biotechnology (Shanghai, China). In addition, aspartate aminotransferase (AST), alanine aminotransferase (ALT), and γ-glutamyltransferase (GGT) activities were analyzed using commercially available kits from Nanjing Jiancheng Bioengineering Institute (Nanjing, China).

#### 2.4.3. Antioxidant and Immune-Related Enzyme Parameters

The activities of superoxide dismutase (SOD), glutathione peroxidase (GSH-Px), catalase (CAT), and lysozyme (LYZ), as well as the levels of immunoglobulin M (IgM) and malondialdehyde (MDA), in serum and liver samples were quantified using commercial assay kits supplied by Shanghai Enzyme-linked Biotechnology Co., Ltd. (Shanghai, China).

#### 2.4.4. Gene Expression Analysis

Primers targeting metabolic enzyme genes were designed from GenBank sequences (Table 2). Total RNA was extracted from liver tissues using the TransZol Up Plus RNA Kit (Beijing TransGen Biotech Co., Ltd., Beijing, China). RNA integrity was confirmed, and RNA concentration and purity were assessed through spectrophotometry (A260:280 nm) following 1% agarose gel electrophoresis. cDNA was synthesized using the PrimeScript™ RT Reagent Kit (Takara Bio Inc., Tokyo, Japan) according to the manufacturer’s protocol. β-actin was selected as the internal reference gene. Real-time PCR was conducted on a LightCycler 480 System (Roche Diagnostics, Rotkreuz, Switzerland) with a reaction volume of 10 μL, containing 5 μL of 2X SYBR^®^ Green Pro Taq HS Premix II (Accurate Biotechnology Co., Ltd., Changsha, China), 3.8 μL of RNase-free water, 1 μL of cDNA, and 0.1 μL each of forward and reverse primers. The thermal cycling protocol consisted of 95 °C for 30 s, followed by 40 cycles of 95 °C for 5 s and 60 °C for 30 s. The relative expression of the target gene was quantified utilizing the 2^−ΔΔCT^ method [22].

### 2.5. Ammonia Nitrogen Stress Challenge

Following the 10-week feeding trial, an ammonia nitrogen stress challenge was conducted using experimental fish from each dietary group. Ammonia nitrogen at varying concentrations was prepared using NH_4_Cl. The 96 h median lethal concentration (LC50) of ammonia nitrogen for largemouth bass was determined through preliminary trials, followed by the formal ammonia stress test. Mortality rates of largemouth bass in different treatment groups were recorded, and the relative protection rate (RPR) was calculated. A 10 g/L NH_4_Cl stock solution was prepared and diluted to the desired ammonia concentrations, which were used immediately. Each experimental tank had a water volume of 60 L. Based on the preliminary trial results, the 96 h LC50 of ammonia nitrogen for largemouth bass was determined to be 95 mg/L, with all test water adjusted and maintained at pH 7.5 ± 0.2 and temperature 25 ± 1 °C. The formal experiment consisted of six treatment groups, each with three replicates, and six fish per replicate. During the stress test, a normal oxygen supply was maintained, and no feeding was performed. Mortality was recorded for each experimental group.

### 2.6. Calculation

$$\mathrm{Weight}\;\mathrm{gain}\;\mathrm{rate}\;\left(\%\right)=100\;\times \;\frac{\mathrm{Wf}\;-\;\mathrm{Wi}}{\mathrm{Wi}}$$

$$\mathrm{Specific}\;\mathrm{growth}\;\mathrm{rate}\;\left(\%/d\right)=100\;\times \;\frac{\mathrm{lnWf}\;-\;\mathrm{lnWi}}{d}$$

$$F\mathrm{eed}\;\mathrm{conversion}\;\mathrm{ratio}=\frac{\mathrm{feed}\;\mathrm{consumption}}{\mathrm{Wf}\;-\;\mathrm{Wi}}$$

$$\mathrm{Protein}\;\mathrm{efficiency}\;\mathrm{ratio}=100\;\times \;\frac{\mathrm{Wf}\;-\;\mathrm{Wi}}{\mathrm{protein}\;\mathrm{intake}}$$

$$\mathrm{Survival}\;\mathrm{rate}\;(\%)=100\;\times \;\frac{\mathrm{final}\;\mathrm{fish}\;\mathrm{number}}{\mathrm{initial}\;\mathrm{fish}\;\mathrm{number}}$$

$$\mathrm{Condition}\;\mathrm{factor}\;(g/{\mathrm{cm}}^{3})=100\;\times \;\frac{\mathrm{body}\;\mathrm{weight}(g)}{{\mathrm{body}\;\mathrm{length}\;(\mathrm{cm})}^{3}}$$

$$\mathrm{Viscerosomatic}\;\mathrm{index}\;(\%)=100\;\times \;\frac{\mathrm{viscera}\;\mathrm{weight}}{\mathrm{whole}\;\mathrm{body}\;\mathrm{weight}}$$

$$\mathrm{Hepatosomatic}\;\mathrm{index}\;(\%)=100\;\times \;\frac{\mathrm{hepatic}\;\mathrm{weight}}{\mathrm{whole}\;\mathrm{body}\;\mathrm{weight}}$$

where Wi and Wf represent the initial and final body weight (g), respectively, and d represents the number of experimental days.

### 2.7. Statistical Analysis

Normality (Shapiro–Wilk test) and homogeneity of variances (Levene’s test) were verified before analysis. Data were analyzed using one-way ANOVA (SPSS 25.0) followed by Tukey’s post hoc test (*p* < 0.05). Values are presented as means ± standard error of the mean (SEM). To evaluate dose–response relationships, orthogonal polynomial contrasts were performed to assess linear and quadratic trends. A linear or quadratic model was considered meaningful only when both the overall ANOVA and the polynomial contrast were significant (*p* < 0.05). The adjusted coefficient of determination (Adj. R^2^) was calculated according to Kvålseth (1985) to indicate the proportion of variance explained by the model [25]; higher Adj. R^2^ values indicate a better model fit. For significant SOP models, the optimal inclusion level (OIL) of LOLA was calculated from the vertex of the parabola.

## 3. Results

### 3.1. Growth Performance

The 10-week trial revealed that dietary LOLA inclusion, as well as supplementation with [L-ornithine + L-aspartic acid], had no significant effect on growth performance (weight gain, specific growth rate), feed utilization (feed conversion rate, protein efficiency ratio), or body indices (condition factor, viscerosomatic index, hepatosomatic index) (*p* > 0.05; Table 3).

### 3.2. Protein Metabolism-Related Indexes

Dietary LOLA supplementation, as well as supplementation with [L-ornithine + L-aspartic acid], had no significant effect on plasma levels of TAA, GH, or IGF-1 (*p* > 0.05; Table 4). However, plasma BUN and INS levels followed SOP responses, increasing initially and then decreasing with increasing LOLA levels, while plasma BA levels showed an SOP trend characterized by an initial decrease followed by an increase. The optimal inclusion levels (OILs) for BUN, INS, and BA were 0.08%, 0.06%, and 0.06%, respectively (*p* < 0.001; *p* < 0.001; *p* = 0.002). Additionally, compared with the OA9 group, the LOLA9 group significantly reduced the plasma BA level (*p* < 0.05).

Dietary LOLA supplementation, as well as supplementation with [L-ornithine + L-aspartic acid], did not significantly affect serum ALT activity, AST/ALT, plasma GDH, and hepatic AMPD activities (*p* > 0.05; Table 5). However, serum AST activity exhibited an SOP trend, with an OIL of 0.08% (*p* = 0.001). Similarly, plasma AMPD and hepatic GDH activities followed SOP trends, decreasing initially and then increasing with increasing LOLA supplementation. Additionally, compared with the OA9 group, the LOLA9 group significantly reduced plasma AMPD and hepatic GDH activities (*p* < 0.05). Conversely, plasma GGT activity exhibited a SOP trend, increasing initially and then decreasing. Furthermore, compared with the OA9 group, the LOLA9 group significantly increased plasma GGT activity (*p* < 0.05). The OILs for plasma AMPD, hepatic GDH, and plasma GGT were 0.07%, 0.08%, and 0.07%, respectively (*p* = 0.008; *p* < 0.001; *p* < 0.001).

### 3.3. Antioxidant and Immune Function-Related Parameters

**Figure 1:**
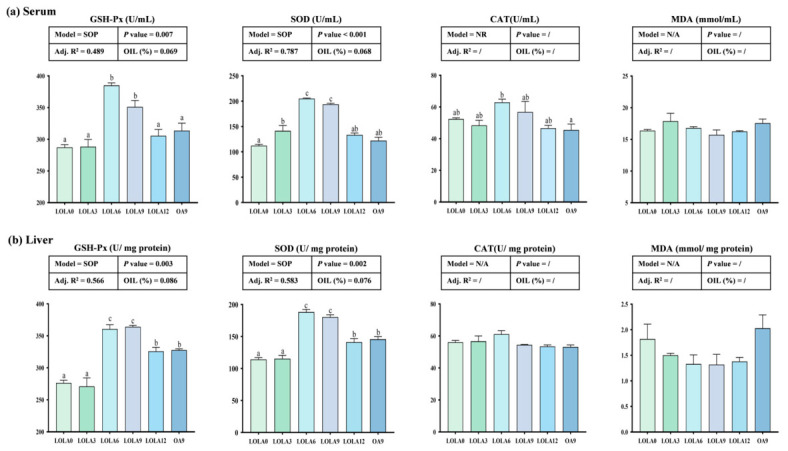
Effect of dietary L-ornithine L-aspartate (LOLA) on antioxidant response of largemouth bass (*Micropterus salmoides*). (**a**) Serum antioxidant enzyme activities; (**b**) Hepatic antioxidant enzyme activities; Values represent means with standard errors shown as vertical bars (*n* = 3). Different lowercase letters above the bars denote significant differences (*p* < 0.05). NR, no relationship; N/A, not applicable; SOP, second-order polynomial trend; Adj. R^2^, adjusted R-squared; OIL, optimal inclusion level of LOLA; /, no valid quadratic fit (OIL not estimable). GSH-Px, glutathione peroxidase; SOD, superoxide dismutase; CAT, catalase; MDA, malondialdehyde.

Dietary LOLA supplementation, as well as supplementation with [L-ornithine + L-aspartic acid], had no significant effect on hepatic CAT activity or serum or hepatic MDA content (*p* > 0.05; Figure 1). However, serum and hepatic GSH-Px and SOD activities followed SOP trends, increasing initially and then decreasing with increasing LOLA levels. The OILs of serum GSH-Px, hepatic GSH-Px, serum SOD, and hepatic SOD were 0.07%, 0.07%, 0.09%, and 0.08%, respectively (*p* = 0.007; *p* < 0.001; *p* = 0.003; *p* = 0.002). Additionally, compared with the OA9 group, the LOLA9 group significantly increased the activities of serum and hepatic GSH-Px and SOD (*p* < 0.05).

With increasing LOLA supplementation, serum and hepatic levels of LYZ and IgM followed SOP trends, increasing initially and then decreasing. The OILs for all four parameters were 0.07% (*p* = 0.005; *p* = 0.004; *p* = 0.003; *p* < 0.001; Table 6). Additionally, compared with the OA9 group, the LOLA9 group significantly increased the levels of serum LYZ and IgM, and hepatic IgM (*p* < 0.05).

### 3.4. The Relative mRNA Expression Levels of Liver Health-Related Genes

**Figure 2:**
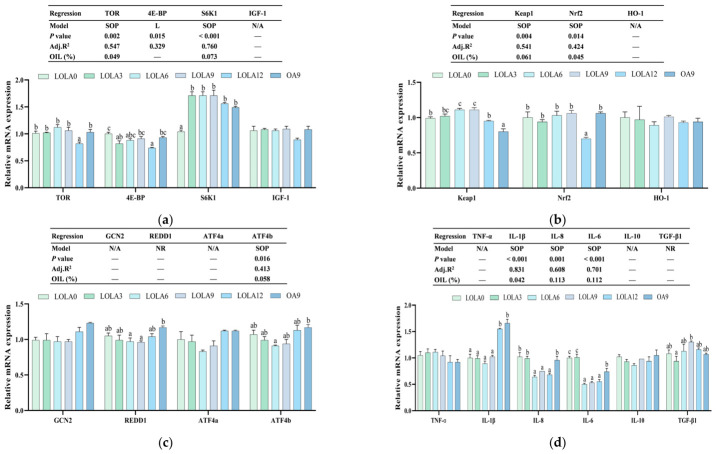
Effects of dietary L-ornithine L-aspartate (LOLA) on the hepatic health of largemouth bass (*Micropterus salmoides*). (**a**) TOR pathway-related genes; (**b**) Antioxidant-related genes; (**c**) AAR pathway-related genes; (**d**) Inflammatory response-related genes. Values represent means with standard errors shown as vertical bars (*n* = 3). Different lowercase letters above the bars denote significant differences (*p* < 0.05). NR, no relationship; N/A, not applicable; SOP, second-order polynomial trend; Adj. R^2^, adjusted R-squared; OIL, optimal inclusion level of LOLA.

Dietary LOLA supplementation, as well as supplementation with [L-ornithine + L-aspartic acid], had no significant effect on the hepatic mRNA expression levels of IGF-1, HO-1, GCN2, ATF4a, TNF-α, or IL-10 (*p* > 0.05; Figure 2). However, the expression levels of TOR, S6K1, Keap1, and Nrf2 followed SOP trends characterized by an initial increase followed by a decrease, with OILs of 0.05%, 0.07%, 0.06%, and 0.05%, respectively (*p* = 0.002; *p* < 0.001; *p* = 0.004; *p* = 0.014; Figure 2a,b). Additionally, compared with the OA9 group, the LOLA9 group significantly increased the expression levels of Keap1 (*p* < 0.05). Conversely, the expression levels of ATF4b, IL-1β, IL-8, and IL-6 followed SOP trends characterized by an initial decrease followed by an increase, with OILs of 0.06%, 0.04%, 0.11%, and 0.11%, respectively (*p* = 0.016; *p* < 0.001; *p* = 0.001; *p* < 0.001; Figure 2c,d). Furthermore, the expression of 4E-BP exhibited a linear trend (*p* = 0.015). Additionally, compared with the OA9 group, the LOLA9 group significantly decreased the expression levels of REDD1, IL-1β, IL-8, and IL-6 (*p* < 0.05).

### 3.5. Mortality and Relative Protection Rate Under Ammonia Nitrogen Stress

Dietary LOLA supplementation, as well as supplementation with [L-ornithine + L-aspartic acid], had no significant effect on the RPR (*p* > 0.05; Table 7). However, the mortality rate followed a SOP trend, decreasing initially and then increasing, with an OIL of 0.064% (*p* = 0.019). Additionally, compared with the OA9 group, the LOLA9 group showed no significant differences in mortality rate and RPR (*p* > 0.05).

## 4. Discussion

Ornithine participates in the urea cycle and primarily facilitates ammonia detoxification, thereby alleviating the toxic effects associated with ammonia accumulation in the body [26,27,28]. Previous studies have indicated that dietary supplementation with 5–20 g/kg ornithine has no significant effect on the growth performance of rainbow trout [29,30]. Conversely, other studies have shown that incorporating 0.6–1.5 g/kg ornithine into the diet significantly enhances weight gain, feed efficiency, and protein deposition in Pacific white shrimp (*Litopenaeus vannamei*) [31]. Similarly, L-aspartic acid, as a functional amino acid, plays a crucial role in the metabolism of mammalian intestinal epithelial cells [32], and dietary L-aspartic acid supplementation has been shown to enhance growth performance and alleviate oxidative stress in pigs [33,34]. Furthermore, LOLA has been reported to increase body weight and muscle mass in mice [35], and dietary supplementation with 400–500 mg/kg LOLA increases body weight and average gain while reducing feed conversion ratio in broilers [36]. Nevertheless, studies on the effects of dietary LOLA on growth performance in aquatic animals remain very limited. In the present study, neither LOLA nor [L-ornithine + L-aspartate] supplementation significantly affected the growth performance or feed utilization of largemouth bass during the 10-week feeding trial. This absence of growth response may be attributable to several factors. First, previous studies on formulated diets for aquatic animals indicate that growth performance is strongly influenced by dietary protein levels [37,38]; under such nutritionally adequate conditions, the scope for further growth enhancement by functional amino acid additives may be limited. Second, the 10-week feeding period may be insufficient for the observed metabolic improvements to translate into measurable growth gains, as such effects may require longer adaptation or become more evident under low-nutrient dietary conditions. Third, it is also possible that the biochemical and molecular changes, although statistically significant, were not of sufficient magnitude to drive detectable improvements in growth performance. We acknowledge that these interpretations remain speculative and warrant further investigation.

Ammonia nitrogen is the primary form of nitrogen excretion in fish and is mainly produced through amino acid catabolism [39,40]. In contrast, urea nitrogen, which is generated through ammonia conversion via the ornithine cycle, serves as a secondary nitrogenous excretory product [41,42]. The level of ammonia nitrogen excretion directly reflects the extent to which fish use amino acids as energy substrates [43]. We observed that nitrogen metabolism-related indicators exhibited coordinated changes. Specifically, dietary supplementation with 0.06% LOLA significantly increased plasma INS levels while concurrently decreasing BA levels. As a key anabolic hormone, elevated INS levels are generally associated with activation of anabolic signaling in the organism [44]. Simultaneously, decreased BA levels may indicate reduced amino acid deamination for energy production. These results are consistent with the hypothesis that LOLA may not only reduce the ammonia nitrogen load requiring urea cycle processing but also conserve more amino acids for protein synthesis. This finding is consistent with previous reports showing that LOLA reduces blood ammonia concentrations and thereby enhances ammonia clearance and metabolism [18]. Additionally, a lower BUN level generally indicates reduced protein catabolism or improved protein utilization efficiency [45]. Correspondingly, according to SOP regression analysis, the OIL for plasma BUN was calculated to be 0.08%. Dietary inclusion of 0.08% LOLA significantly decreased plasma BUN levels, collectively suggesting that 0.06–0.08% LOLA supplementation was associated with changes in nitrogen metabolism and amino acid utilization. However, we acknowledge that these biomarker changes reflect altered metabolic status rather than direct evidence of enhanced protein deposition, and further studies are needed to confirm whether these changes translate into functional outcomes. The ammonia-sparing effect of LOLA observed here may be mediated by the hepatic glutamate–glutamine cycle. Fish adapt to ammonia stress through urea/glutamine conversion pathways, with glutamine synthetase playing a critical role in ammonia detoxification [46]. In broilers, LOLA supplementation up-regulated glutamine synthetase (GS) and down-regulated GDH, thereby channeling ammonia into glutamine rather than generating urea or free ammonia [36]. Although GS activity was not directly measured in our study, the significant reduction in hepatic GDH activity and plasma BA levels may suggest a similar mechanism in largemouth bass. However, we acknowledge that this interpretation remains speculative in the absence of direct measurements of GS activity or ammonia assimilation pathways, and further studies are needed to confirm the involvement of the glutamate–glutamine cycle in the responses observed here. Notably, no significant difference in plasma TAA levels was observed among the dietary groups. This unchanged TAA level, together with reduced BA and elevated INS, suggests that LOLA was associated with changes in amino acid utilization patterns, redirecting amino acids toward protein synthesis rather than simply increasing the circulating amino acid pool. Similarly, although plasma GH and IGF-1 remained unchanged, hepatic TOR and S6K1 expression were significantly upregulated by LOLA supplementation, which may suggest that LOLA was associated with activation of intracellular anabolic signaling at the transcriptional level. Importantly, compared with supplementation with [L-ornithine + L-aspartate], dietary LOLA significantly reduced the BA level, suggesting a more pronounced change in ammonia-related metabolites. LOLA is a stable salt comprising ornithine and aspartate, and has been reported to facilitate ammonia clearance by stimulating urea cycle activity and glutamine synthesis in mammalian systems [47,48]. However, as direct measurements of these pathways were not performed, the mechanistic basis for the observed difference between LOLA and the separate amino acid mixture remains to be established. The more pronounced changes observed with LOLA compared with separate supplementation of its constituent amino acids may reflect differential absorption, metabolic availability, or coordinated nitrogen disposal; nevertheless, this interpretation remains speculative and warrants further investigation.

Nitrogen metabolism-related enzymes are essential indicators of fish health and nutritional status. Among these enzymes, ALT and AST play important roles in nitrogen assimilation in fish [11]. In addition, hepatic GDH and AMPD are involved in nitrogen metabolism and ammonia production, respectively [49]. Specifically, AMPD participates in the purine nucleotide cycle, and reduced AMPD activity may decrease ammonia production [50]. GDH is a key enzyme linking amino acid metabolism to the tricarboxylic acid cycle [51]. Both GDH and ALT are regarded as indicators of amino acid utilization [52]. In this work, the OILs for plasma AMPD, hepatic GDH, and AST activities were 0.07%, 0.07%, and 0.08%, respectively. These results may suggest that dietary supplementation with 0.07–0.08% LOLA was associated with changes in amino acid utilization efficiency and nitrogen metabolic processes. The serum AST/ALT ratio is considered a reliable indicator of liver damage [53], and an elevated ratio has been associated with liver injury in rats [54]. It should be noted that dietary LOLA supplementation significantly reduced serum AST activity, but no significant changes were observed in serum ALT activity or the AST/ALT ratio. Since ALT and AST are commonly used markers of hepatic cell integrity [55], the unaltered ALT level and AST/ALT ratio suggest that LOLA supplementation did not induce hepatocellular injury, supporting the safe use of LOLA in largemouth bass. Although the differences were not statistically significant, numerically lower AST/ALT ratios were observed in the LOLA6 and LOLA9 groups compared with the control group. This result is consistent with previous findings indicating that LOLA enhances ammonia clearance and metabolism by modulating serum transaminase activity [18]. Furthermore, GGT primarily catalyzes glutathione degradation and γ-glutamyl transfer. This function links GGT activity to antioxidant status and amino acid transport [56]. In the present study, compared with supplementation with [L-ornithine + L-aspartate], dietary LOLA supplementation significantly reduced plasma AMPD and hepatic GDH activities, suggesting that LOLA was associated with greater reductions in ammonia production and accumulation. Regarding the markedly higher GGT activity observed in the OA9 group, it is unlikely to reflect hepatocellular injury, as the AST/ALT ratio showed no significant changes. Instead, GGT is involved in glutathione metabolism and amino acid transport across cell membranes [56]. The elevated GGT in the OA9 group may reflect an adaptive metabolic response to separate amino acid supplementation, possibly involving enhanced glutathione turnover or amino acid transport. The mammalian target of rapamycin (mTOR) is a central regulator of cell growth and metabolism, integrating signals from nutrients and growth factors to control protein synthesis [57,58]. The present study further showed that appropriate LOLA doses regulated key protein synthesis pathways at the molecular signaling level. Specifically, dietary supplementation with 0.05–0.07% LOLA significantly upregulated hepatic TOR expression and its downstream effector S6K1, which was interpreted as modulation of TOR-S6K1-related markers. These transcriptional changes may be associated with ribosome biogenesis and translation initiation at the molecular level [59], but direct evidence for enhanced protein deposition was not obtained. Furthermore, the AAR pathway is activated under amino acid deficiency, during which GCN2 may inhibit translation initiation and suppress the TOR pathway through ATF4-mediated signaling, ultimately inhibiting protein synthesis and cell growth [60,61]. The expression of ATF4b, a key downstream effector of the AAR pathway, was significantly down-regulated by 0.06% LOLA supplementation. Regarding GCN2 and ATF4a, no significant differences were detected, although a decreasing trend in ATF4a expression was observed in the LOLA6 and LOLA9 groups. GCN2 functions as the upstream sensor of amino acid deficiency, and its unaltered expression indicates that the cells did not perceive a shortage of amino acids. The numerical downregulation of ATF4a, together with the significant suppression of ATF4b, suggests a consistent tendency toward reduced AAR pathway activity. This down-regulation may indicate that LOLA alleviated cellular amino acid deprivation signals and that the AAR pathway was not activated but rather suppressed at the optimal dose. These findings suggest that LOLA may have enabled the organism to utilize amino acids more efficiently for anabolic processes rather than diverting them toward stress-adaptive responses [62]. Further studies are needed to confirm whether these molecular modifications translate into increased protein synthesis or deposition. In addition, although LOLA9 and OA9 did not differ significantly in promoting protein synthesis, the consistently lower BA and hepatic GDH activities in the LOLA9 group suggest that LOLA may be associated with greater nitrogen-sparing and ammonia detoxification responses than in the final growth output under the present experimental conditions. However, no significant difference was observed between the LOLA9 and OA9 groups. Additionally, the RPR among all groups did not differ significantly. This lack of difference warrants cautious interpretation due to several experimental limitations. First, the challenge test was conducted with only six fish per replicate and three replicates per treatment, which limited the statistical power to detect small to moderate differences in mortality. Second, the mortality rate in the control group (72.22%) substantially exceeded the expected 50% at the theoretical LC_50_, likely due to inaccuracies in ammonia nitrogen preparation, fluctuations in pH and temperature, and batch-to-batch variation among fish. Third, the acute ammonia challenge, while providing a standardized and reproducible stress model, does not fully reflect the chronic or fluctuating ammonia exposure conditions typically encountered in commercial aquaculture. Nevertheless, because the RPR was calculated using the same control group, this deviation did not affect relative comparisons among the treatment groups. The mortality data still serve as a useful reference, and the optimal LOLA dose for ammonia resistance derived from the SOP regression of mortality data (approximately 0.06%) aligns with the OILs for other physiological parameters, suggesting a coherent dose-dependent effect that warrants further investigation. Future studies with larger sample sizes and longer observation periods are needed to validate these findings.

Key enzymes, including CAT, SOD, and GSH-Px, play critical roles in the antioxidant defense system of fish [63]. These enzymes effectively scavenge and neutralize free radicals, including reactive oxygen species, thereby mitigating oxidative damage in fish [64]. In addition, MDA serves as a reliable biomarker of lipid peroxidation [65]. In mammals, dietary aspartate supplementation has been shown to reduce reactive oxygen species (ROS) levels and enhance antioxidant enzyme activity by improving nitrogen metabolism [66]. Importantly, dietary supplementation with 500 mg/kg in broilers significantly increased the activities of GSH-Px, SOD, CAT, and T-AOC in both the liver and serum, while decreasing the levels of MDA and ROS [67]. The present study demonstrated that dietary supplementation with an appropriate dose of LOLA was associated with changes in both antioxidant defense parameters and nonspecific immune indicators in largemouth bass. Specifically, the OILs for serum GSH-Px and SOD, hepatic GSH-Px and SOD were 0.07%, 0.09%, 0.07%, and 0.08%, respectively. Notably, although no significant differences were detected in MDA content in either serum or liver, a numerical decrease in hepatic MDA was observed in the LOLA6 and LOLA9 groups compared with the control group; the lack of statistical significance may be attributable to statistical reasons. Correspondingly, supplementation with 0.05–0.06% LOLA significantly upregulated the hepatic expression of Keap1 and Nrf2. It is possible that the enhanced antioxidant capacity observed here may be related to improved mitochondrial function, although mitochondrial parameters were not directly assessed. In broilers, LOLA has been reported to increase mitochondrial membrane potential and oxygen consumption rate, thereby reducing electron leakage and ROS production [67]. The observed upregulation of Nrf2 and Keap1 expression here is consistent with activation of the endogenous antioxidant defense system, which can be triggered by mitochondrial stress signaling. However, this interpretation remains speculative, as mitochondrial function was not directly measured in our study, and further investigation is needed to confirm this potential mechanism. Thus, LOLA may protect against ammonia-induced mitochondrial dysfunction in fish liver, a hypothesis that warrants further investigation. Regarding immunity, when organisms are exposed to harmful substances, humoral immune responses, such as LYZ activity, are activated before cytokine responses [68]. IgM functions as the primary immunoglobulin in fish [69]. Furthermore, numerous studies have demonstrated that fish immune status can be assessed based on the balance between anti-inflammatory factors, such as IL-10 and TGF-β, and pro-inflammatory factors, such as IL-8, IL-1β, and TNF-α. In broilers, LOLA reduced serum IL-1β and TNF-α but increased IL-10, attenuating systemic inflammation. At 42 d, LOLA also down-regulated hepatic IL-1β and TNF-α mRNA and up-regulated IL-10, alleviating liver inflammation [67]. LYZ and IgM levels peaked at 0.07% LOLA, whereas the expression of pro-inflammatory cytokines, including IL-1β, IL-8, and IL-6, was significantly suppressed in our study. These results suggest that LOLA supplementation may be associated with modulation of certain immune-related parameters, including enhanced humoral immune indicators and reduced expression of pro-inflammatory cytokines. However, anti-inflammatory cytokines such as IL-10 remained unaffected, indicating that the immune response was partial rather than a systemic enhancement. This finding is consistent with previous reports demonstrating that LOLA modulates inflammatory response factors [18]. In summary, dietary inclusion of 0.05–0.08% LOLA was associated with changes in antioxidant and immune parameters in largemouth bass, but further studies are needed to confirm whether these changes represent a systematic enhancement of immune defense. Moreover, compared with supplementation with [L-ornithine + L-aspartate], dietary LOLA supplementation showed more pronounced changes in several antioxidant and immune parameters. However, as these differences were not consistently observed across all endpoints and direct measurements of functional immune outcomes were not performed, these findings should be interpreted as suggestive of differential physiological responses. In summary, although no significant growth-promoting effects were observed during the 10-week feeding period, dietary supplementation with 0.05–0.08% LOLA was associated with changes in several physiological indicators, including modulation of nitrogen metabolism, antioxidant and immune parameters, and alterations in the expression of genes associated with protein synthesis. These findings suggest that, in aquatic animals, LOLA may exert more pronounced effects on selected physiological and molecular markers than on growth performance under the present experimental conditions. Based on the current market price (approximately 530 RMB/kg), LOLA supplementation at 0.05–0.08% would add approximately 265–424 RMB per ton of feed. Whether this cost is justified would depend on the frequency of ammonia-related stress episodes in commercial operations, and further economic analysis is needed to determine the cost-effectiveness threshold for largemouth bass producers. In addition, further studies are warranted to elucidate the full range of functional roles and underlying mechanisms of LOLA in aquatic species, including its potential effects on mitochondrial function.

## 5. Conclusions

In conclusion, dietary inclusion of 0.05–0.08% LOLA was associated with changes in ammonia-related metabolites, antioxidant parameters, and immune indicators in largemouth bass without compromising growth performance. Mechanistically, LOLA supplementation was associated with upregulation of TOR S6K1-related markers, Nrf2/Keap1-mediated antioxidant gene expression, and suppression of AAR-related markers and pro-inflammatory cytokine expression. Notably, LOLA at 0.06% showed a significant reduction in mortality under acute ammonia stress. Compared with 0.09% L-ornithine + L-aspartate (OA9), LOLA showed more pronounced changes in several ammonia-related metabolites as well as antioxidant and immune parameters. These findings provide preliminary evidence for the effects of dietary LOLA in fish and suggest its potential as a feed additive for further investigation; however, confirmatory studies are needed to establish its functional benefits and practical application in intensive aquaculture of largemouth bass.

## Figures and Tables

**Table 1:** Formulation and proximate composition of experimental diets (dry matter, %).

Ingredient	LOLA0	LOLA3	LOLA6	LOLA9	LOLA12	OA9
Brown fishmeal	24.00	24.00	24.00	24.00	24.00	24.00
Poultry by-product meal	8.00	8.00	8.00	8.00	8.00	8.00
Soybean meal	26.60	26.60	26.60	26.60	26.60	26.60
Cottonseed protein concentrate	16.00	16.00	16.00	16.00	16.00	16.00
Wheat flour	10.00	10.00	10.00	10.00	10.00	10.00
Soybean oil	4.10	4.10	4.10	4.10	4.10	4.10
Fish oil	3.00	3.00	3.00	3.00	3.00	3.00
Soybean lecithin	1.50	1.50	1.50	1.50	1.50	1.50
L-Ornithine L-Aspartate	0.00	0.03	0.06	0.09	0.12	0.00
L-Aspartate	0.00	0.00	0.00	0.00	0.00	0.045
L-Ornithine hydrochloride	0.00	0.00	0.00	0.00	0.00	0.057
Microcrystalline cellulose	4.00	3.97	3.94	3.91	3.88	3.898
Vitamin and mineral premix ^1^	0.70	0.70	0.70	0.70	0.70	0.70
Others ^2^	2.10	2.10	2.10	2.10	2.10	2.10
Proximate composition						
Dry matter (DM, %)	87.57	86.95	88.54	88.68	88.19	88.76
Crude protein (% DM)	48.83	48.71	48.39	48.59	48.85	48.60
Crude lipid (% DM)	11.30	11.14	11.63	11.60	11.45	11.61
Ash (% DM)	9.22	9.19	9.38	9.37	9.39	9.50

^1^ Vitamin and mineral premix (g/kg of mixture): vitamin A, 0.20 g; vitamin D3, 0.003 g; vitamin E, 4.40 g; vitamin K_3_, 0.66 g; vitamin B_1_, 0.33 g; vitamin B_2_, 0.88 g; vitamin B_6_, 0.73 g; vitamin B_12_, 0.001 g; nicotinic acid, 2.89 g; calcium pantothenate, 1.64 g; folic acid, 0.07 g; biotin, 0.003 g; vitamin C, 10.01 g; FeSO_4_·7H_2_O, 52.87 g; H_3_ClCu_2_O_3_, 0.65 g; ZnSO4·7H_2_O, 43.15 g; MnSO_4_·7H_2_O, 31.56 g; MgSO_4_·H_2_O, 44.65 g; Ca(IO_3_)_2_, 0.42 g; Na_2_SeO_3_, 0.11 g; CoCl_2_·6H_2_O, 0.14 g. ^2^ Others included 1.50% Ca(H_2_PO_4_)_2_, 0.20% NaCl, 0.35% choline chloride, 0.02% vitamin C, and 0.03% ethoxyquin. All proximate composition values were determined experimentally.

**Table 2:** Primer sequence used for qPCR analysis.

Gene	Primer Sequence (5′–3′)	Source
β-actin	F: GGACACGGAAAGGATTGACAG R: CGGAGTCTCGTTCGTTATCGG	XM_047442803.1
TOR	F: CCAAAGACGTGCTGTTCACC R: GAGCCTTCAGAAACCTGCGA	XM_038702468
4E-BP	F: ACGAGGTCTGCCCAACATTC R: CAGCGTTGCTGCTATCAGGT	XM_038703879.1
S6K1	F: GCCAATCTCAGCGTTCTCAAC R: CTGCCTAACATCATCCTCCTT	XM_038708508.1
IGF-1	F: CTTCAAGAGTGCGATGTGC R: GCCATAGCCTGTTGGTTTACTG	[23]
GCN2	F: GAATATCGTCCGCTACTACA R: CGTCGTCGTCATCATCAT	XM_038728156.1
REDD1	F: TGACCTGTGTCCCTCTAATGA R: ATGTGCTCCAGAAGTTTCTCA	XM_038735581.1
ATF4a	F: GCCTCCGCTTCCCTCTCCTC R: CTGCCGTCTTGTTCTGCTCCATC	XM_038712790.1
ATF4b	F: GAGAGTGAGGTGAAGCCAGTTGTG R: GGAGCCAGAACGAGGACAATGC	XM_038695435.1
Keap1	F: GTGGTGGGAAGACTTATTG R: TCCAGGTGCTTAGTGAGG	XM_038728593.1
Nrf2	F: TCACCAAAGACAAGCGTAA R: CAGGCAGATTGATAATCATAGA	XM_038720536.1
HO-1	F: ATCGGAGCAGATTAAGGC R: TTGTACTGTGGCAGGGTG	XM_038694281.1
TNF-α	F: CTTCGTCTACAGCCAGGCATCG R: TTTGGCACACCGACCTCACC	XM_038710731.1
IL-1β	F: CGTGACTGACAGCAAAAAGAGG R: GATGCCCAGAGCCACAGTTC	NM_001244959.2
IL-8	F: CGTTGAACAGACTGGGAGAGATG R: AGTGGGATGGCTTCATTATCTTGT	XM_038704088.1
IL-6	F: GACTGGAGTGGCGGAAAGTGGAGG R: TTTCATCTTCTACAAACGCAGACAACGG	[24]
IL-10	F: CGGCACAGAAATCCCAGAGC R: CAGCAGGCTCACAAAATAAACATCT	NM_013438.5
TGF-β1	F: GCTCAAAGAGAGCGAGGATG R: TCCTCTACCATTCGCAATCC	NM_001412790.1

β-actin, beta-actin; TOR, target of rapamycin; 4E-BP, eukaryotic initiation factor 4E-binding protein; S6K1, S6 kinase 1; IGF-1, insulin-like growth factor 1; GCN2, general control nondepressible 2; REDD1, regulated in development and DNA damage response 1; ATF4a, activating transcription factor 4a; ATF4b, activating transcription factor 4b; Keap1, kelch-like ECH-associated protein 1; Nrf2, nuclear factor erythroid 2-related factor 2; HO-1, heme oxygenase-1; TNF-α, tumour necrosis factor-alpha; IL-1β, interleukin-1 beta; IL-8, interleukin-8; IL-6, interleukin-6; IL-10, interleukin-10; TGF-β1, transforming growth factor beta 1.

**Table 3:** Effect of dietary L-ornithine L-aspartate (LOLA) on growth performance of largemouth bass (*Micropterus salmoides*).

Ingredient	LOLA0	LOLA3	LOLA6	LOLA9	LOLA12	OA9
Final body weight (g)	72.34 ± 0.71	71.31 ± 2.02	72.85 ± 0.14	71.52 ± 1.34	69.45 ± 3.08	71.47 ± 1.74
Weight gain rate (%)	884.69 ± 12.52	870.85 ± 30.06	885.45 ± 5.54	869.00 ± 14.10	835.82 ± 39.64	864.20 ± 26.91
Specific growth rate (%/d)	3.22 ± 0.02	3.20 ± 0.04	3.22 ± 0.01	3.20 ± 0.02	3.15 ± 0.06	3.19 ± 0.04
Feed conversion ratio	1.15 ± 0.01	1.16 ± 0.05	1.15 ± 0.03	1.15 ± 0.01	1.12 ± 0.03	1.15 ± 0.03
Protein efficiency ratio	1.77 ± 0.02	1.77 ± 0.07	1.81 ± 0.05	1.78 ± 0.01	1.84 ± 0.05	1.79 ± 0.04
Survival rate (%)	91.11 ± 1.11	86.66 ± 3.33	87.78 ± 2.22	90.00 ± 0.00	88.89 ± 2.22	90.00 ± 1.92
Condition factor (g/cm^3^)	2.16 ± 0.04	2.27 ± 0.10	2.16 ± 0.04	2.14 ± 0.08	2.05 ± 0.05	2.06 ± 0.04
Viscerosomatic index (%)	7.03 ± 0.32	6.94 ± 0.21	7.24 ± 0.26	7.31 ± 0.29	7.23 ± 0.22	7.06 ± 0.18
Hepatosomatic index (%)	1.10 ± 0.05	1.10 ± 0.14	1.15 ± 0.09	1.18 ± 0.07	1.16 ± 0.13	1.10 ± 0.10

Values are expressed as means ± standard error of the means (*n* = 3).

**Table 4:** Effect of dietary L-ornithine L-aspartate (LOLA) on protein metabolism-related indices in plasma of largemouth bass (*Micropterus salmoides*).

	Diets						Regression			
	LOLA0	LOLA3	LOLA6	LOLA9	LOLA12	OA9	Model	*p* Value	Adj. R^2^	OIL (%)
TAA (μmol/mL)	83.44 ± 1.74	82.23 ± 0.92	82.80 ± 1.48	83.26 ± 0.67	83.56 ± 1.28	83.34 ± 0.66	N/A	/	/	/
BUN (nmol/L)	93.59 ± 0.64 ^b^	89.16 ± 2.15 ^ab^	86.15 ± 0.83 ^a^	87.08 ± 1.22 ^a^	87.31 ± 1.75 ^a^	87.93 ± 0.36 ^a^	SOP	0.004	0.545	0.083
BA (μmol/L)	589.39 ± 7.51 ^e^	554.97 ± 5.98 ^c^	503.03 ± 3.10 ^a^	531.03 ± 2.17 ^b^	580.63 ± 6.18 ^de^	565.31 ± 1.53 ^cd^	SOP	<0.001	0.828	0.064
INS (mU/L)	56.01 ± 1.00 ^a^	58.77 ± 0.47 ^ab^	61.99 ± 0.52 ^b^	58.90 ± 0.44 ^ab^	57.28 ± 1.25 ^ab^	56.28 ± 1.88 ^a^	SOP	0.002	0.578	0.064
GH (ng/mL)	28.82 ± 0.13	28.93 ± 0.22	28.35 ± 0.58	29.09 ± 0.14	28.87 ± 0.63	28.49 ± 0.30	N/A	/	/	/
IGF-1 (ng/mL)	29.61 ± 0.02	28.80 ± 0.40	29.06 ± 0.38	27.87 ± 1.90	27.63 ± 0.96	26.88 ± 0.14	N/A	/	/	/

Values are expressed as means ± standard error of the means (*n* = 3). Means within the same row with different superscripts indicate statistically significant differences among treatments (*p* < 0.05). N/A, not applicable; SOP, second-order polynomial trend; Adj. R^2^, adjusted R-squared; OIL, optimal inclusion level of LOLA; /, no valid quadratic fit (OIL not estimable). TAA, total amino acids; BUN, blood urea nitrogen; BA, blood ammonia; INS, insulin; GH, growth hormone; IGF-1, insulin-like growth factor-1.

**Table 5:** Effect of dietary L-ornithine L-aspartate (LOLA) on protein metabolism-related enzyme activities of largemouth bass (*Micropterus salmoides*).

	Diets						Regression			
	LOLA0	LOLA3	LOLA6	LOLA9	LOLA12	OA9	Model	*p* Value	Adj. R^2^	OIL (%)
Serum										
AST (U/L)	13.37 ± 0.50 ^b^	10.70 ± 0.67 ^a^	10.01 ± 0.93 ^a^	9.45 ± 0.15 ^a^	10.80 ± 0.27 ^a^	10.38 ± 0.60 ^a^	SOP	0.001	0.669	0.076
ALT (U/L)	7.57 ± 1.12	6.41 ± 0.21	6.38 ± 0.26	6.53 ± 0.22	6.26 ± 0.27	7.13 ± 0.29	N/A	/	/	/
AST/ALT	1.82 ± 0.18	1.68 ± 0.16	1.57 ± 0.11	1.45 ± 0.07	1.73 ± 0.10	1.46 ± 0.04	N/A	/	/	/
Plasma										
AMPD (U/mL)	1.08 ± 0.01 ^b^	1.06 ± 0.03 ^b^	0.86 ± 0.02 ^a^	0.84 ± 0.07 ^a^	1.03 ± 0.01 ^b^	1.02 ± 0.00 ^b^	SOP	0.008	0.480	0.071
GDH (U/L)	16.06 ± 0.05	15.16 ± 0.41	14.78 ± 0.26	14.70 ± 0.19	15.66 ± 1.02	14.64 ± 0.23	N/A	/	/	/
GGT (U/L)	5.87 ± 0.29 ^a^	8.12 ± 0.38 ^b^	9.27 ± 0.67 ^b^	9.55 ± 0.58 ^b^	8.45 ± 0.25 ^b^	15.42 ± 0.20 ^c^	SOP	<0.001	0.762	0.078
Liver										
AMPD (U/mg protein)	0.88 ± 0.04	0.91 ± 0.02	0.88 ± 0.02	0.64 ± 0.02	0.90 ± 0.03	0.81 ± 0.13	N/A	/	/	/
GDH (U/g protein)	14.93 ± 0.14 ^c^	11.65 ± 0.06 ^b^	8.91 ± 0.55 ^a^	9.59 ± 0.64 ^a^	13.36 ± 0.38 ^c^	13.35 ± 0.18 ^c^	SOP	<0.001	0.891	0.066

Values are expressed as means ± standard error of the means (*n* = 3). Means within the same row with different superscripts indicate statistically significant differences among treatments (*p* < 0.05). N/A, not applicable; SOP, second-order polynomial trend; Adj. R^2^, adjusted R-squared; OIL, optimal inclusion level of LOLA; /, no valid quadratic fit (OIL not estimable). AST, aspartate aminotransferase; ALT, alanine aminotransferase; AMPD, adenosine monophosphate deaminase; GDH, glutamate dehydrogenase. GGT, gamma-glutamyl transferase.

**Table 6:** Effect of dietary L-ornithine L-aspartate (LOLA) on immune-related indices of largemouth bass (*Micropterus salmoides*).

	Diets						Regression			
	LOLA0	LOLA3	LOLA6	LOLA9	LOLA12	OA9	Model	*p* Value	Adj. R^2^	OIL (%)
Serum										
LYZ (U/mL)	0.24 ± 0.02 ^b^	0.24 ± 0.01 ^b^	0.40 ± 0.01 ^c^	0.41 ± 0.01 ^c^	0.24 ± 0.01 ^b^	0.20 ± 0.01 ^a^	SOP	0.005	0.521	0.067
IgM (mg/mL)	1.28 ± 0.03 ^a^	1.25 ± 0.09 ^a^	2.29 ± 0.04 ^c^	2.08 ± 0.03 ^b^	1.31 ± 0.03 ^a^	1.30 ± 0.05 ^a^	SOP	0.004	0.529	0.067
Liver										
LYZ (U/mg protein)	0.27 ± 0.02 ^a^	0.32 ± 0.03 ^ab^	0.39 ± 0.01 ^c^	0.36 ± 0.01 ^bc^	0.31 ± 0.01 ^ab^	0.31 ± 0.01 ^ab^	SOP	0.003	0.559	0.067
IgM (μg/mg protein)	1.40 ± 0.05 ^a^	1.83 ± 0.08 ^b^	2.11 ± 0.05 ^c^	1.94 ± 0.06 ^bc^	1.83 ± 0.04 ^b^	1.32 ± 0.06 ^a^	SOP	0.000	0.818	0.073

Values are expressed as means ± standard error of the means (*n* = 3). Means within the same row with different superscripts indicate statistically significant differences among treatments (*p* < 0.05). SOP, second-order polynomial trend; Adj. R^2^, adjusted R-squared; OIL, optimal inclusion level of LOLA. LYZ, lysozyme; IgM, immunoglobulin M.

**Table 7:** Effect of dietary L-ornithine L-aspartate (LOLA) on resistance to ammonia nitrogen challenge in largemouth bass (*Micropterus salmoides*).

	Diets						Regression			
	LOLA0	LOLA3	LOLA6	LOLA9	LOLA12	OA9	Model	*p* Value	Adj. R^2^	OIL (%)
Mortality rate (%)	72.22 ± 5.56 ^b^	61.11 ± 5.56 ^ab^	38.89 ± 5.56 ^a^	55.56 ± 5.56 ^ab^	66.67 ± 9.62 ^ab^	55.56 ± 5.56 ^ab^	SOP	0.019	0.396	0.064
RPR (%)	/	15.38 ± 7.69	46.16 ± 7.69	23.08 ± 7.69	7.69 ± 13.32	23.08 ± 7.69	N/A	/	/	/

Values are expressed as means ± standard error of the means (*n* = 3). Means within the same row with different superscripts indicate statistically significant differences among treatments (*p* < 0.05). SOP, second-order polynomial trend; N/A, not applicable; Adj. R^2^, adjusted R-squared; OIL, optimal inclusion level of LOLA; /, no valid quadratic fit (OIL not estimable). Mortality rate = 100 × (initial fish number − final fish number)/initial fish number; RPR, relative protection rate = 100 × (mortality of control group − mortality of treatment group)/mortality of control group.

## Data Availability

Data will be made available on request.

## Abbreviations

The following abbreviations are used in this manuscript:

LOLA	L-Ornithine L-aspartate
TAA	Total Amino Acids
BUN	Blood Urea Nitrogen
BA	Blood Ammonia
INS	Insulin
GH	Growth Hormone
IGF-1	Insulin-Like Growth Factor 1
AMPD	Adenosine Monophosphate Deaminase
GDH	Glutamate Dehydrogenase
AST	Aspartate Aminotransferase
ALT	Alanine Aminotransferase
SOD	Superoxide Dismutase
GSH-Px	Glutathione Peroxidase
CAT	Catalase
LYZ	Lysozyme
IgM	Immunoglobulin M
MDA	Malondialdehyde
RPR	Relative Protection Rate
SOP	Second-Order Polynomial
GS	Glutamine Synthetase
ROS	Reactive Oxygen Species
